# Asymptomatic COVID-19 Infection-Induced Rhabdomyolysis in the Backdrop of Statin-Cyclosporine Drug Interaction

**DOI:** 10.7759/cureus.68127

**Published:** 2024-08-29

**Authors:** Vivek Nayak M, FNU Bipasha, Kotresha Neelakantappa

**Affiliations:** 1 Internal Medicine, Kasturba Medical College, Manipal, Manipal, IND; 2 Internal Medicine, Government Medical College and Hospital, Chandigarh, IND; 3 Internal Medicine, MedStar Union Memorial Hospital, Baltimore, USA; 4 Nephrology, New York Presbyterian Brooklyn Methodist Hospital, Brooklyn, USA

**Keywords:** creatine kinase, myoglobin, chronic kidney disease, cyclosporine, statin, acute kidney injury, rhabdomyolysis, covid-19

## Abstract

Rhabdomyolysis involves skeletal muscle breakdown leading to high serum creatine kinase (CK) levels and myoglobinuria. Here, we report the case of a middle-aged man who developed rhabdomyolysis, resulting in acute kidney injury (AKI) over pre-existing chronic kidney disease (stage 3a) secondary to focal segmental glomerulosclerosis (primary FSGS), during an asymptomatic COVID-19 infection. The patient had been on treatment with cyclosporine and statin, among other drugs, for his comorbidities. He had initially presented to the hospital after a fall due to difficulty walking in the setting of increasing edema. Lab workup revealed elevated CK and AKI. Urinalysis showed “large” blood on a dipstick with only two RBCs per high-power field on microscopy, suggesting myoglobinuria. A standard respiratory pathogen polymerase chain reaction panel revealed positive SARS-CoV-2. The chest X-ray and oxygenation were normal, and he had no respiratory symptoms. He was treated with intravenous fluids and albumin, with a steady improvement in renal function. Our case underlines that rhabdomyolysis can occur in asymptomatic COVID-19 infection. Therefore, it may be worth monitoring CK levels in COVID-19-positive patients with risk factors for rhabdomyolysis, such as the concurrent usage of statins and cyclosporine, even if they are otherwise asymptomatic.

## Introduction

Rhabdomyolysis refers to the lysis/breakdown of skeletal muscle cells or rhabdomyocytes with the release of their intracellular constituents into circulation, such as myoglobin, enzymes (creatine kinase (CK), transaminases, lactate dehydrogenase), and electrolytes (potassium, phosphate) [1-3]. Its causes may be physical, such as trauma/crush injury, prolonged immobilization, intense muscle activity (seizure, exertion, neuroleptic malignant syndrome), burns, and electrocution. It can have non-physical causes such as drugs, toxins, venoms, infections, genetic myopathies, dyselectrolytemia, ischemia, and more. These non-physical causes induce muscle damage by direct injury to the sarcolemma, adenosine triphosphate depletion, and other metabolic mechanisms [2,3]. Drugs like statins and colchicine cause direct myotoxicity, whereas cocaine and amphetamines cause vasoconstrictive ischemia [4,5]. Concurrent usage of statins with other drugs like steroids, cyclosporine, gemfibrozil, and CYP450 inhibitors in general increases the risk of statin-induced rhabdomyolysis. Of the many complications of rhabdomyolysis, pigment (myoglobin)-induced acute kidney injury (AKI) is common, seen in up to 30% of cases, especially so when CK levels are more than 5000 IU/L [2].

COVID-19 is a known infectious cause of rhabdomyolysis; however, the literature suggests that usually, only severe COVID-19 leading to hypoxia causes rhabdomyolysis due to an energy supply-demand mismatch [1]. Often, other risk factors are concurrently present with severe COVID-19 that may contribute to this, such as old age, diabetes, hypertension, hypothyroidism, injury, inborn errors of metabolism, and use of myotoxic drugs like statins, fluoroquinolones, macrolide antibiotics, HIV protease inhibitors, antipsychotics, and chemotherapy agents [1,6]. Notably, the SARS CoV-2 virus spike protein is known to cause host cell mitochondrial dysfunction, leading to altered bioenergetics and cellular breakdown in muscles [1]. Asymptomatic COVID-19-associated rhabdomyolysis and AKI is a less reported occurrence [7]. This article will discuss this rare scenario, focusing on underlying risk factors.

## Case presentation

A middle-aged male in his early 50s presented to the ED after a fall. He had a past medical history that was significant for nephrotic syndrome secondary to focal segmental glomerulosclerosis (FSGS), on cyclosporine therapy with partial response, chronic kidney disease (CKD) stage 3a, hypoalbuminemia, hypothyroidism, hyperlipidemia, epilepsy, disorganized schizophrenia, and bipolar disorder. He reported that he had chronic bilateral lower extremity swelling due to his kidney disease, which had been worsening for the last two weeks, leading to difficulty with ambulation. He said that he was going to the hospital and fell while going past the door. The ambulance was called, and he was brought to the ED. He was conscious and moving at that time. There was no major injury, head strike, tongue biting, or urinary incontinence. His last reported seizure was two to three years ago, and he endorsed taking all medications as prescribed. He denied any dark urine or decreased urine output. He denied fever, chills, night sweats, cough, sore throat, chest pain, or myalgia. There was no history of smoking, alcohol consumption, or intravenous drug use. The patient had a family history of cardiovascular disease.

The patient's home medications included atorvastatin for hyperlipidemia, cyclosporine for steroid and cyclophosphamide-resistant nephrotic syndrome secondary to FSGS, antiepileptics (divalproex, levetiracetam, lacosamide, and lamotrigine), levothyroxine for hypothyroidism, psychiatric medications for schizophrenia (aripiprazole, risperidone, and benztropine), and enalapril for proteinuria. He was last seen in the renal clinic more than a month ago when his latest urine protein/creatinine ratio was 2,758, which had fallen significantly from 4,237 about nine months ago. Of note, his cyclosporine dose was increased by 25% a month before admission due to subtherapeutic levels in the blood, after which it had attained therapeutic levels for a while.

On arrival at the emergency department, the patient was afebrile and hemodynamically stable, with a physical exam notable for 3+ pitting edema of bilateral lower extremities up to the hip with mild tenderness, chronic scrotal edema, flat abdomen, and chest clear to auscultation. Other systemic examinations were within normal limits.

Investigations

Initial laboratory investigations (Table 1) were suggestive of mild normocytic anemia with hemoglobin of 12.7 g/dL (baseline 12-13), AKI on CKD (creatinine = 3.52 mg/dL, baseline of 1.6-1.8), elevated serum CK (>22,000 IU/L), and BNP >3,000 pg/mL. Liver function tests showed marked transaminitis and hypoalbuminemia. In addition, he had an elevated ESR. Thyroid function testing revealed an elevated thyroid-stimulating hormone (TSH) with normal T3 and T4. Urinalysis showed cloudy-orange appearing urine with dipstick “large” positive for blood but only two RBCs per high power field, a disparity highly suggestive of myoglobinuria versus hemoglobinuria. The former was more likely given elevated CK and transaminases, raising suspicion for rhabdomyolysis. Overall, his laboratory values were significant for rhabdomyolysis and AKI on CKD. However, serum electrolytes and arterial blood gas analysis were normal.

**Table 1:** Initial laboratory investigations H: high, L: low, AST: aspartate transaminase, ALT: alanine transaminase, ALP: alkaline phosphatase, WBCs: white blood cells, ESR: erythrocyte sedimentation rate, AST: aspartate transaminase, ALT: alanine transaminase, ALP: alkaline phosphatase, TSH: thyroid-stimulating hormone, CK: creatine kinase

Laboratory investigation (units)	Reference range	Patient values
Complete blood picture		
Hemoglobin (g/dL)	13.7-17.5	12.7 (L)
Hematocrit (%)	40.1-51.0	38.2 (L)
Platelets (/mm^3^)	150,000-450,000	170,000
WBCs (/mm^3^)	3,400-11,200	3,880
Inflammatory markers		
ESR (mm/hr)	0-20	124 (H)
Renal function tests and electrolytes		
Urea (mg/dL)	6-20	57 (H)
Creatinine (mg/dL)	0.67-1.17	3.53 (H)
Sodium (mmol/L)	136-145	138
Potassium (mmol/L)	3.5-5.1	4.7
Chloride (mmol/L)	98-107	106
Bicarbonate (mmol/L)	22-29	26
Calcium (mmol/L)	8.6-10.0	8.6
Liver function tests		
Total protein (g/dL)	6.6-8.7	4.0 (L)
Albumin (g/dL)	3.5-5.2	<1.0 (L)
Globulin (g/dL)	1.5-5.2	2.7
Total bilirubin (mg/dL)	≤1.2	<0.2
AST (U/L)	10-50	1768 (H)
ALT (U/L)	10-50	576 (H)
ALP (U/L)	40-129	122
Thyroid function tests		
TSH (µIU/mL)	0.3-4.2	5.8 (H)
Free T_4_ (ng/dL)	0.93-1.70	0.88 (L)
Other enzymes		
CK, total (IU/L)	≤190	>22,000 (H)

**Figure 1:**
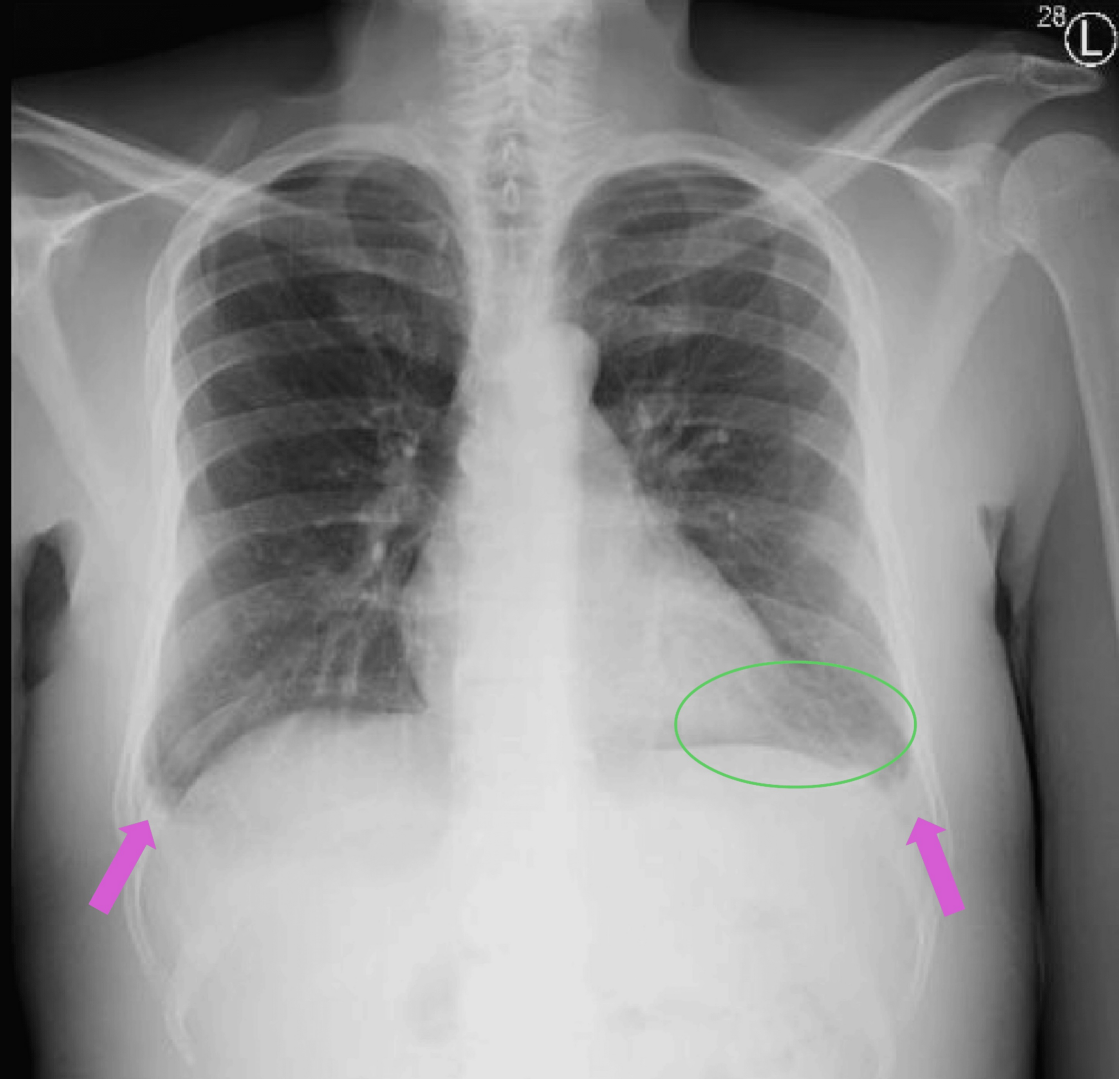
Chest X-ray Chest radiograph showing no evidence of pneumonia. Also seen are discoid atelectasis in the left lung base (green circle) and blunting of the costophrenic angles (pink arrows), possibly due to minimal effusion. The remaining lung fields show no active infiltrates or consolidations.

The patient also underwent further workup of etiology for rhabdomyolysis. The urine drug screen was negative. EEG was normal, and seizures were ruled out based on history. On routine respiratory pathogen panel testing for infectious etiology, he was found to be COVID-19 PCR positive on a nasopharyngeal swab. However, serial chest X-rays (Figure 1) were negative for pneumonia. Given the absence of fever, cough, or chest X-ray findings and normal oxygen requirements, this was an asymptomatic COVID-19 infection. Given the pedal edema, a lower extremity duplex scan was performed and was negative for deep vein thrombosis. In addition, transthoracic echocardiography showed normal cardiac function, and ECG showed a normal sinus rhythm. Further cardiac evaluation was deferred since the elevated BNP was presumed to be due to impaired excretion in the setting of renal dysfunction. Liver ultrasound showed normal echogenicity. Renal ultrasound showed increased echogenicity of the right kidney, with normal echogenicity of the left kidney. Therapeutic drug testing of blood showed elevated levels of levetiracetam (93 mcg/mL; range 10-40), subtherapeutic levels of valproate (29.1 mcg/mL, range 50-100), and subtherapeutic levels of cyclosporine (35.7 ng/mL; range 100-400).

Treatment

**Figure 2:**
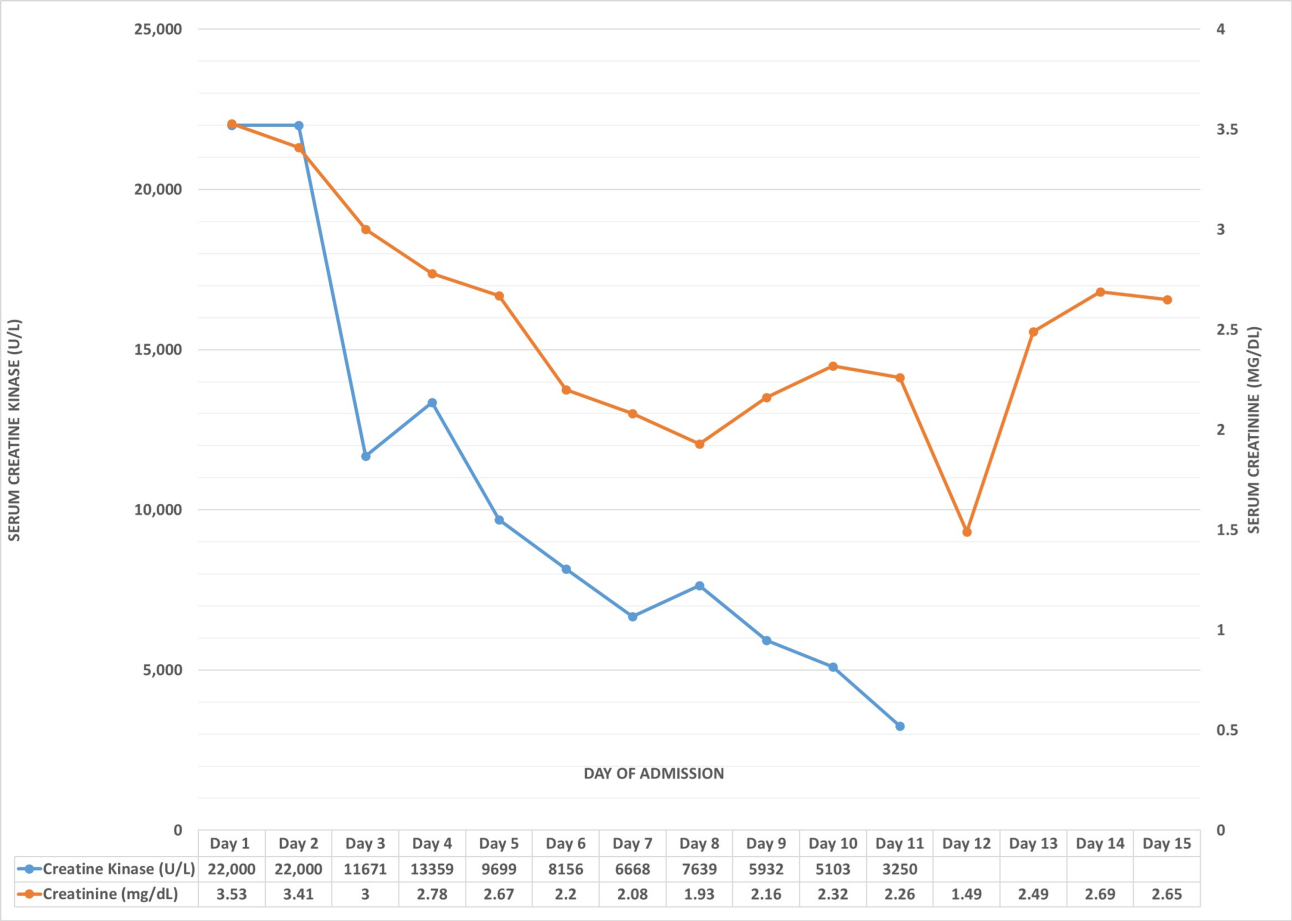
Trends in CK and creatinine levels Line graph showing the daily trends in serum CK and serum creatinine levels. While CK shows an overall downward trend, creatinine values initially fall with ongoing intravenous hydration but rise on day 13 as a response to additional diuretics to reduce edema, eventually falling again. CK: creatine kinase

The patient was treated with IV fluids and IV 25% albumin in order to restore intravascular volume and manage AKI. Renal function gradually improved, with serum creatinine, CK, and transaminases trending down (Figure 2). Serum albumin levels also improved subsequently. Urine output was normal throughout this admission. Given that the patient was polyuric and in the recovery phase of AKI, additional IV fluids were discontinued and oral fluids were started. IV albumin supplementation was continued to maintain intravascular volume and renal function, CK, and transaminases further trended toward the baseline. ESR also declined. Also, given elevated TSH, his dose of levothyroxine was increased.

Outcome and follow-up

Over the course of treatment, the patient developed increasing edema due to overhydration. However, he was treated with diuretics at the expense of some increase in creatinine, with edema eventually subsiding to his baseline. He never had any respiratory symptoms or oxygen requirements throughout this admission. He received physical therapy to assist with ambulation. Eventually, he was discharged to a rehabilitation facility. He has been well since then and had no new complaints on his last follow-up four months later.

## Discussion

Patients with rhabdomyolysis usually present with myalgia, weakness, dark urine (myoglobinuria), and prodromal systemic symptoms like fever and malaise [2]. Rhabdomyolysis, the only feature of an otherwise asymptomatic COVID-19 infection, is rare. This occurrence has been described in the adolescent population in the absence of pre-existing risk factors for rhabdomyolysis [7,8]. In a case described by Tram et al., an otherwise healthy adolescent developed rhabdomyolysis and renal failure in the setting of COVID-19 infection without any respiratory symptoms [7]. However, similar cases in the adult population, such as ours, could not be found in the literature. The diagnosis of rhabdomyolysis is made by consistent clinical features and serum CK >1,000 U/L. Laboratory investigations may also reveal raised AST and ALT, serum potassium, and phosphate, all released from lysed myocytes. Hypocalcemia can occur secondary to intravascular calcium-phosphate binding. However, this patient did not have any electrolyte abnormalities. Increased creatinine above the baseline indicates pigment-induced AKI, which this patient had [2,3]. A diagnosis of myoglobinuria is suggested by a dipstick positive for blood (by detecting myoglobin), but no RBCs on microscopy, and simultaneously elevated CK and transaminases in the blood (to differentiate from hemoglobinuria) [9].

Rhabdomyolysis is managed by aggressive intravenous fluid resuscitation to prevent or treat AKI, with careful titration to the urine output. It may be prudent to consider the volume status of the patient as well. In this particular case, it can be stated retrospectively that over-hydration should have been prevented, given the patient was already volume-overloaded at the time of presentation. Intravenous albumin supplementation alone may have been sufficient to increase intravascular volume. Whenever CK levels are more than 30,000 IU/L, forced alkaline diuresis with sodium bicarbonate is a good option, whereby the urine is alkalinized to prevent precipitation of myoglobin in the distal convoluted tubules. Treatment of the cause and treating electrolyte abnormalities are also important [2,10,11].

The patient, in this case, had a few risk factors for the development of rhabdomyolysis, such as medications (atorvastatin, levetiracetam, and antipsychotics), statin-cyclosporine interaction resulting in elevated statin levels, and hypothyroidism. He also had hypoalbuminemia secondary to FSGS, which is a poor prognostic factor, increasing the risk of AKI in rhabdomyolysis [3]. The patient had no documented evidence of rhabdomyolysis in the past. Furthermore, the potential triggers he had for rhabdomyolysis were injury due to the fall, possible seizure, and the incidentally discovered COVID-19 infection. However, the likely etiology is the COVID-19 infection, and in the trailing discussion, we have tried to rule out all other possible triggers.

The fall episode as described by the patient did not sound seizure-related, and he did not recount any recent episodes of seizure. CK levels in rhabdomyolysis secondary to seizure activity peak within 24-72 hours, and the rate of decline is also relatively constant, decreasing by about 39% of the previous day’s value in a regular downward trend [5]. In this patient, the CK was already >22,000 at admission, suggesting that it was either at peak or had peaked before admission. Two days after the fall, CK was 11,671 and rose to 13,539 the day after and then dropped by 27.4% in a day to 9,699, suggesting an irregular pattern. Furthermore, the rhabdomyolysis was unlikely to have been caused by the fall itself, as the patient neither sustained significant muscle injury nor was he immobilized for long. Most cases of rhabdomyolysis, severe enough to cause AKI, are seen in patients who fall unconscious and are unable to move for many hours and develop enough trauma to the muscles just from body weight. This patient sustained minimal trauma from the fall without being immobile on the floor for any length of time. Also, high levels of CK were seen on the same day as the fall and thereafter only trended downwards. Muscle injury induces a rise-peak-fall type of CK pattern, hence making it unlikely to be the trigger here [2]. The patient had also complained of increasing bilateral lower extremity swelling with pain over a few days before admission, suggesting that rhabdomyolysis likely predates his admission. In addition, his raised ESR with a subsequent falling trend points more toward infection versus an inflammatory state. Thus, COVID-19 was the most likely trigger for rhabdomyolysis.

Of the risk factors predisposing the patient to rhabdomyolysis, atorvastatin with concomitant use of cyclosporine appears the most important. Statins are well-known to cause a spectrum of myopathies, ranging from myalgia to rhabdomyolysis [12]. The risk of rhabdomyolysis with statin is usually within one month after its onset or when the dose is intensified. Our patient had been taking the same dose of statin for many years. However, considering that he is also on cyclosporine, which has pharmacokinetic interactions with statins in the form of increased statin levels, the risk is increased more than with statin alone. Also, his cyclosporine dose was increased a month before his admission, given the subtherapeutic levels. Atorvastatin has been reported to cause rhabdomyolysis when used along with cyclosporine, and its pharmacokinetic and pharmacological properties make such a drug interaction likely [13]. Levetiracetam may also cause rhabdomyolysis as one of its rare side effects, but it’s usually observed within three days of initiation of the drug [14,15]. This patient has been taking levetiracetam for years. However, levetiracetam levels were found to be elevated in this patient, almost double the therapeutic maximum, which could be due to impaired excretion in the setting of AKI. Rhabdomyolysis is a rare complication of hypothyroidism but is only seen if the patient is not adherent to treatment, which is not the case here [16]. Lastly, the patient was on antipsychotics, which are known to cause rhabdomyolysis even in the absence of neuroleptic malignant syndrome [17,18]. Both aripiprazole and risperidone have been implicated in this regard [19,20]. However, the patient had been taking them for a few years; hence, they are unlikely to be the cause of rhabdomyolysis.

## Conclusions

Overall, this is likely a case of rhabdomyolysis triggered by an otherwise asymptomatic COVID-19 infection. Yet, it is important to note that COVID-19-induced rhabdomyolysis has occurred in the backdrop of several risk factors, most significantly statin-cyclosporine drug interaction. This pharmacokinetic interaction leads to elevated plasma levels of statin, thus risking skeletal muscle damage. COVID-19 may have just been the trigger that pushed statin-induced myopathy toward full-blown rhabdomyolysis.

Rhabdomyolysis and AKI may be the only presenting features of COVID-19 infection. Also, it may be worth monitoring CK levels in those patients who test positive for COVID-19, especially if they have pre-existing risk factors or symptoms of rhabdomyolysis. Early initiation of intravenous fluid therapy is the key to management, both to prevent and treat myoglobin-induced AKI.

## References

[1] Preger A, Wei R, Berg B, Golomb BA (2023). COVID-19-associated rhabdomyolysis: a scoping review. Int J Infect Dis.

[2] Stanley M, Chippa V, Aeddula NR, Rodriguez BSQ, Adigun R (2024). Rhabdomyolysis. StatPearls [Internet].

[3] Giannoglou GD, Chatzizisis YS, Misirli G (2007). The syndrome of rhabdomyolysis: pathophysiology and diagnosis. Eur J Intern Med.

[4] Valiyil R, Christopher-Stine L (2010). Drug-related myopathies of which the clinician should be aware. Curr Rheumatol Rep.

[5] Khan FY (2009). rhabdomyolysis: a review of the literature. Neth J Med.

[6] Bawor M, Sairam S, Rozewicz R, Viegas S, Comninos AN, Abbara A (2022). Rhabdomyolysis after COVID-19 infection: a case report and review of the literature. Viruses.

[7] Tram N, Chiodini B, Montesinos I (2020). Rhabdomyolysis and acute kidney injury as leading COVID-19 presentation in an adolescent. Pediatr Infect Dis J.

[8] Gilpin S, Byers M, Byrd A, Cull J, Peterson D, Thomas B, Jacobson P (2021). Rhabdomyolysis as the initial presentation of SARS-CoV-2 in an adolescent. Pediatrics.

[9] Anwar MY, Gupta V (2024). Myoglobinuria. StatPearls [Internet].

[10] Zutt R, van der Kooi AJ, Linthorst GE, Wanders RJA, de Visser M (2014). Rhabdomyolysis: review of the literature. Neuromuscul Disord.

[11] Hebert JF, Burfeind KG, Malinoski D, Hutchens MP (2023). Molecular mechanisms of rhabdomyolysis-induced kidney injury: from bench to bedside. Kidney Int Rep.

[12] Abed W, Abujbara M, Batieha A, Ajlouni K (2022). Statin induced myopathy among patients attending the National Center for Diabetes, Endocrinology, and Genetics. Ann Med Surg (Lond).

[13] Maltz HC, Balog DL, Cheigh JS (1999). Rhabdomyolysis associated with concomitant use of atorvastatin and cyclosporine. Ann Pharmacother.

[14] Moinuddin IA (2020). Suspected levetiracetam-induced rhabdomyolysis: a case report and literature review. Am J Case Rep.

[15] Kubota K, Yamamoto T, Kawamoto M, Kawamoto N, Fukao T (2017). Levetiracetam-induced rhabdomyolysis: a case report and literature review. Neurol Asia.

[16] Salehi N, Agoston E, Munir I, Thompson GJ (2017). Rhabdomyolysis in a patient with severe hypothyroidism. Am J Case Rep.

[17] Packard K, Price P, Hanson A (2014). Antipsychotic use and the risk of rhabdomyolysis. J Pharm Pract.

[18] Tiglis M, Hurmuzache T, Bologa C, Neagu TP, Mirea L, Grintescu IM (2020). Rhabdomyolysis-induced acute renal injury in a schizophrenic patient. J Crit Care Med (Targu Mures).

[19] Kutlu A, Songür ÇY, Apa H (2023). Aripiprazole-associated rhabdomyolysis in a 17-year-old male. Arch Iran Med.

[20] Look ML, Boo YL, Chin PW, Hoo FK (2017). Risperidone-associated rhabdomyolysis without neuroleptic malignant syndrome: a case report. J Clin Psychopharmacol.

